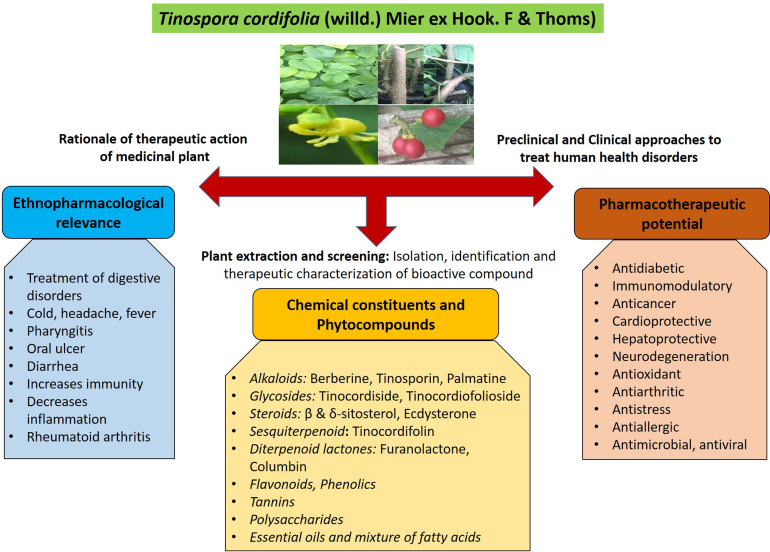# *Tinospora cordifolia (Giloy)*: An insight on the multifarious pharmacological paradigms of a most promising medicinal ayurvedic herb

**DOI:** 10.1016/j.heliyon.2024.e26125

**Published:** 2024-02-15

**Authors:** Abhishek Gupta, Priyanka Gupta, Gunjan Bajpai

**Affiliations:** a Baj's Laboratories, Industrial Area, Rooma, Kanpur-208008, UP, India; b King George's Medical University, Lucknow-226003, UP, India

**Keywords:** *Tinospora cordifolia*, Medicinal herb, Chemical compounds, Phytoconstituents, Immunity, Pharmacological activities

## Abstract

Medicinal herbs are being widely accepted as alternative remedies for preventing various diseases especially in India and other Asian countries. However, most plant-based herbal medicines are not yet being scientifically accepted worldwide. “*Tinospora cordifolia* (Willd.) Miers ex Hook.F. & Thomson”, one of the most promising plant species of *Tinospora* known as “Giloy” or Guduchi that is used in several traditional medicines in treating diseases e.g., metabolic and immune disorders, diabetes, heart diseases, cancer, and infectious diseases, has been widely investigated. Varieties of bioactive phytochemical constituents isolated from the stem, root and whole plant of *T. cordifolia* have been identified. In the last two decades, the diverse pharmacological activities of *T. cordifolia* have been continuously studied. Due to its therapeutic efficacy in immune modulation, it could be effective in viral and other diseases treatment as well. A medicinal plant could be well-suited not only for the treatment of target site but also for boosting the body's immune system. As an alternate source of medication, medicinal herbs are continuously showing better compatibility with the human body with minimal side effects than other therapies. Keeping this in mind, the present review highlights the pharmacological potential of *T. cordifolia* against various diseases.

## Highlights

• *T. cordifolia* have anti-diabetic, antioxidant,cardioprotective, hepatoprotective, anti-microbial and other pharmacological activities.
• *T. cordifolia* exerts anti-viral effects through improve platelet count, stimulate macrophages and reduce pro-inflammatory response of cytokines etc.
• *T. cordifolia* could fight against coronavirus disease by boosting immune system, targeting gene and signaling pathways.
• *T. cordifolia* could be the best ayurvedic herbal candidate for drug development in coronavirus and other deadly diseases treatment.
• *T.cordifoli*a could be used as a safe therapeutic herbal medicine against various diseases.

## Abbreviations

ALP: Alkaline Phosphatase
Bcl-xL: B-Cell Lymphoma-Extralarge Protein
CA: Chronic Alcoholism
CamKII-α: Ca2+/Calmodulin-Dependent Protein Kinase II-α
CaN: Calcineurin
CNS: Central Nervous System
COVID-19: Coronavirus Disease-2019
DEN: Diethylnitrosamine
DMARDs: Disease-Modifying Anti-Rheumatic Drugs
DMBA: 7,12-Dimethylbenz(*a*)anthracene
DND: Degenerative Nerve Disease
DNP: Dintrophenyl
DPPH: 1-Diphenyl-2-Picrylhydrazyl
DTD: DT-Diaphorase
EAC: Ehrlich Ascites Carcinoma
EPM: Elevated Plus Maze
GAP-43: Growth Associated Protein-43
GM-CSF: Granulocyte Monocyte-Colony Stimulating Factor
GPx: Glutathione Peroxidase
GR: Glutathione Reductase
GST: Glutathione S-transaminase
HCC: Hepatocellular Carcinoma
HIV: Human Immunodeficiency Virus
IL: Interleukin
LDH: Lactate Dehydrogenase
LDL: Low Density Lipoprotein
l-DOPA: Levodopa
LOX/COX: Lipo-oxygenase/Cyclo-oxygenase
LPS: Lipopolysaccharide
MHC-1: Major Histocompatibility Complex-1
NO: Nitric Oxide
NOR: Novel Object Recognition
NSAIDs: Nonsteroidal Anti-Inflammatory Drugs
6-OHDA: 6-Hydroxydopamine
PEC: Peritoneal Exudate Cells
PMN: Polymorphonuclear
PPAR-α: Peroxisome Proliferator-Activated Receptor-α
PSA-NCAM: Polysialylated Neuronal Cell Adhesion Molecule
RA: Rheumatic Arthritis
ROS: Reactive Oxygen Species
SARS-CoV-2: Severe Acute Respiratory Syndrome Coronavirus 2
SGOT: Serum Glutamic Oxaloacetic Transaminase
SGPT: Serum Glutamic Pyruvate Transaminase
SOD: Superoxide Dismutase
STZ: Streptozotocin
T. cordifolia: Tinospora cordifolia
TBARS: Thiobarbituric Acid Reactive Substances
TG: Triglyceride
TIMP-1: Tissue Inhibitor of Metalloprotease-1
TLR-4: Toll-Like Receptor 4
TNF-α: Tumor Necrosis Factor-α
VEGF: Vascular Endothelial Cell Growth Factor
WBC: White Blood Cells

## 1 Introduction

Asian countries have a wide variety of plants, with enormous floristic diversity in terms of medicinal plants. A large number of medicinal plants that belong to different plant families are being used in medicine for therapeutic purposes to treat many diseases. Medicinal plants capture a vital sector for a healthy society, especially in India, and represent a major natural resource. There are many indigenous systems popular worldwide, such as Ayurveda, Yoga, Siddha, Homeopathy, Unani, and Naturopathy, existing in India and other Asian countries, Africa, Australia and many more. Ayurveda, The Science of Life, describes various medicinal plants in the most refined literature in Sanskrit, Hindi, and regional languages.

*Tinospora* plant has huge potential to treat different diseases. It has been one of the most widely investigated and broadly used medicinal plant in the treatment of various ailments like heart disease, diabetes, leprosy, rheumatoid arthritis, and allergy [1]. It is known as a panacea for almost all the diseases and disorders. *Tinospora* has continuously drawn much more attention from researchers worldwide since the Covid-19 pandemic because of its use as a herbal medicine in primary healthcare and as a home remedy for preventing various diseases and disorders. Medicinal plants such as *Tinospora* have a broad range of applications, ranging from clinical to phytochemistry and pharmacological studies. However, the reverse approach validates scientific output by means of adopting a modern research process known as “reverse pharmacology”.

A total of 34 scientific plant species of the genus *Tinopora* were recorded, of which thirteen were accepted as species names. Three specific medicinal plant species of *Tinospora* (*Cordifolia, Crispa* and *Sinensis)* exist, especially in India. All these species of *Tinospora* belong to the family Menispermaceae of the group Angiosperm. These plant species bear a close resemblance to their visual identification and phytochemical properties of plant parts such as stems, flowers, roots, and leaves. They are widely used for therapeutic purposes in Asia, Africa, and Australia. A previous study revealed that *Tinospora* species were clinically investigated for diabetes, urinary infections, fever, cold, skin inflammation, fungal, and bacterial infections [2].

*Tinospora* species have a broad range of phytochemicals and therapeutic approaches to several diseases. The therapeutic approach of the *Tinospora* plant is attributed to the presence of phytochemical constituents such as alkaloids, flavonoids, glycosides, aliphatic compounds, diterpenoid, vitamins, tannins, lactones, steroids, coumarins, lignans, triterpenes, and nucleosides [3,4]. Out of the three *Tinospora* plant species, *Cordifolia* has much more importance due to its medicinal properties and several therapeutic activities. *T. cordifolia* is helpful in relieving stress and anxiety and has immunomodulatory properties. *T. cordifolia* plant has the potential to inhibit free radical generation and thus protect membranes from radical-induced membrane damage. It is also useful in Dengue because it helps to increase the platelet count. Besides, it has many unknown health benefits and uses. *T. cordifolia* extracts have been used to fight autoimmune disease. It reduces pro-inflammatory cytokines, i.e., interleukin-6 (IL-6), and tumor necrosis factor-α (TNF-α) production in a rheumatoid arthritis rat model [5]. Further, the various extract fractions and ingredients of *T. cordifolia* exhibit antitumor activities [6].

Amongst other ayurvedic medicinal plants, *T. cordifolia* has huge potential to be proven as a highly valued plant with medicinal, ethnopharmacological, phytochemical, and endless properties (Supplement material). Previous studies revealed that *T. cordifolia* has tremendous therapeutic medicinal properties showing anti-diabetic, anti-inflammatory, anti-arthritic, antioxidant, hepatoprotective, cardioprotective, anti-allergic, anti-stress and many more [[6], [7], [8]]. However, detailed studies are further required to elucidate the regulatory pathways to validate the therapeutic potential of *T. cordifolia*. In this focused review, we will detail *T. cordifolia*'s diverse pharmacological activities, which in turn promote science, healthcare, and public awareness of the plant's possible medical benefits.

## 2 Plant description

*Tinospora*, a large deciduous, glabrous perennial plant, is extensively spread throughout India, especially in the tropical parts up to 1.2 km above sea level. It is found in neighboring countries such as China, Sri Lanka, Bangladesh, Pakistan, and Burma [9]. *Tinospora* plants are mostly grown in warm climates. *Tinospora* prefers medium-black or red soil for its cultivation. It can also be successfully grown in a large variety of soils, ranging from sandy to clay loam. However, the soil should be well drained with sufficient moisture and rich with organic matter for its growth.

*T. cordifolia* is commonly known as the Guduchi, Giloy, Amrita, and heart-leaved moonseed plant [10]. It is supposed to be the ambrosia of God Indra, considered a holy liquid. Another species of *Tinospora*, T. *crispa*, a small herb, locally known as Faridbel, is a woody, lofty and entirely glabrous climber. This widely grows herb is found in temperate as well as tropical region of India. Third species, *T. sinensis*/*malabarica* also known as Malabar gulbel, have giant deciduous climber, shining light colored stem, long orbicular-cordate leaves larger than *T. cordifolia*, dioecious flowers, and aerial roots from branches [11].

## 3 Morphological features of *T. cordifolia*

**Fig. 1:**
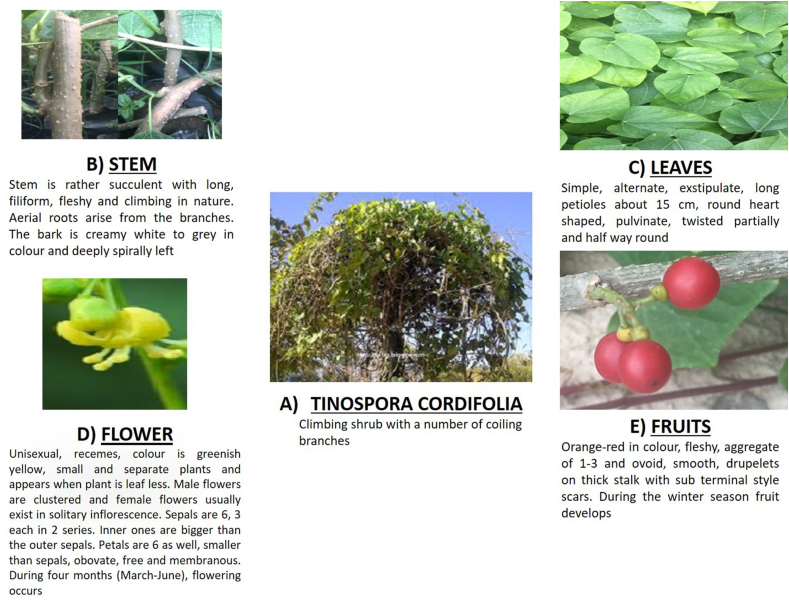
*T. cordifolia* plant and its different parts; a) whole plant; b) stem; c) leaves; d) flowers; and e) fruits. *T. cordifolia* is a large, deciduous, climbing shrub widely used in Ayurvedic system of medicine. Other parts, like the root, dry bark, and lamina, provided important taxonomic characters to the plant. The *T. cordifolia* plant has its own kind of treatment activity. Stem enriches the blood, root reduces stress, and dry bark decreases inflammation and allergic reactions.

*T. cordifolia*, also known as the queen of all herbs, is a climbing shrub with a number of coiling branches. The whole plant has been divided into different parts i.e., stem, leaves, flower, and fruits (Fig. 1). Other parts, like arial roots, lamina, and seeds, are present as well. Tetra-to penta-arch structures are the characteristics of aerial roots. Other than that, the root cortex has an outer, thick wall and an inner parenchymatous zone. The lamina is ovate, 10–20 cm long and up to 15 cm broad; the base is deeply cordate, membranous, pubescent, and whitish tomentose with a prominent reticulum beneath [11]. Seeds are curved in shape. The embryo turned into a curve automatically. However, the endocarp is well ornamented and confers vital taxonomic characters.

## 4 Ethnopharmacological importance of *T. cordifolia*

*T. cordifolia* has a long history of ethnopharmacological importance in traditional medicine systems, particularly in South Asia. *T. cordifolia* stem is mainly used for bitterish, stomachic, astringent, allays thirst, vomiting, burning sensation, enriches the blood, diuretic, thermogenic, stimulates bile secretion, and prevents constipation and jaundice. Its stem has also been considered as indigenous sources of medicines with anti-diabetic, immunomodulatory, anti-hepatotoxic, and antipyretic actions. The stem extract of *T. cordifolia* is effective in the treatment of skin disorders. Another important part of the plant, its root possesses anti-ulcer and anti-stress activity. Both the stem and root of *T. cordifolia*, together with other medicinal drugs, are prescribed as an anti-dote against scorpion sting and snakebite. Dry barks of *T. cordifolia* have anti-inflammatory, antiallergic, antipyretic, antispasmodic, and antileprotic properties.

Several studies published on *T. cordifolia* revealed that it has numerous uses in Ayurvedic medicinal systems. *T. cordifolia* is renowned for its immunomodulatory properties. It is used to enhance the body's natural defense mechanisms and is often recommended to boost the immune system [12,13]. It has been traditionally used to manage various types of fever, including viral and bacterial infections [14,15]. It is believed to help reduce fever symptoms and support the body's fight against pathogens. The plant is known for its antioxidant and anti-inflammatory effects. It is used to alleviate inflammation-related conditions such as arthritis and to counter oxidative stress in the body [5]. *T. cordifolia* is used to improve digestion, reduce acidity and promote overall digestive wellness. It also supports liver health and protect the liver from damage [16,17]. *T. cordifolia* is used to manage diabetes too. It is believed to help regulate blood sugar levels and improve insulin sensitivity. The plant's anti-inflammatory and anti-microbial properties make it useful for managing skin conditions like eczema, psoriasis, and various skin infections. It also alleviates respiratory problems such as asthma, bronchitis, and coughs. It is thought to have bronchodilator and anti-asthmatic effects [18,19]. In Ayurveda, *T. cordifolia* is classified as an adaptogen, or Rasayana, which means it is believed to enhance vitality, reduce stress, and promote overall well-being. There is growing interest in its anticancer properties [20,21]. Research suggests that it may have cytotoxic effects on cancer cells and could be explored further for cancer therapy. Some traditional uses include *T. cordifolia* for cognitive health and its neuroprotective effects [22]. It may be used to enhance memory and protect the nervous system [23]. *T. cordifolia* is often used as a general health tonic in traditional medicine. It is believed to promote longevity, improve vitality and enhance overall health.

## 5 Phytochemical constituents of *T. cordifolia*

*T. cordifolia* is known to contain a wide range of essential chemical constituents, including alkaloids, glycosides, steroids, flavonoids, phenols, tannins, terpenoids, polysaccharides, essential oils, and a combination of fatty acids, all of which have been isolated during preliminary screening. These crucial primary phytoconstituents of *T. cordifolia* are the source of active phytochemical compounds such as b-sitosterol, clerodane furano diterpene, columbin, tinosporine, tinosporide, tinosporaside, cordifolide, cordifol, heptacosanol, and furano diterpene [8]. While the structure of major active phytochemical compounds is displayed in several articles [8,24], a few essential chemical constituents are included in Table 1. All these phytoconstituents have different biological roles and importance and have already been reported in different disease conditions [[25], [26], [27]]. *T. cordifolia* plant material is exhaustively extracted in different forms, such as aqueous [12], methanol [20], ethanol [16], hydro-alcoholic [28], n-hexane [29], chloroform [30,31], and ethyl acetate [32]. Various analytical processes are applied to different extracts of the *T. cordifolia* plant to identify the primary phytoconstituents contained in the sample.

**Table 1:** Phytochemical constituents and active compounds isolated from *T*. *cordifolia*.

Chemicals	Active phytoconstituents	Plant source
Alkaloids	Berberine, Palmatine	Stem
	Tembetarine, Magnoflorine, Choline, Tinosporin, Isocolumbin, Palmatine, Tetrahydropalmatine, Magnoflorine	Root
Glycosides	Tinocordifolioside, Tinocordiside, Cordifoliside A, B, C, D & E, Cordioside, Cordifolioside A & B, Syringin, Syringin-apiosylglycoside, Palmatosides C & F	Stem
Diterpenoid lactones	Furanolactone, Tinosporides, Tinosporon, Jateorine, Columbin, Clerodane derivatives and [(5R,10R)-4R-8R-dihydroxy-2S-3R:15,16-diepoxy-cleroda-13 (16), 14-dieno-17,12S: 18,1S-dilactone]	Whole plant
Steroids	β -sitosterol, δ-sitosterol, 20 β- Hydroxy ecdysone	Aerial part
	Ecdysterone, Makisterone A, Giloinsterol	Stem
Sesquiterpenoid	Tinocordifolin	Stem
Aliphatic compound	Octacosanol, Heptacosanol	Whole plant
Miscellaneous	Tinosporidine, Tinosporic acid, Cordifol, Cordifelone	Whole plant
	Giloin, Giloinin, Jatrorrhizine	Root

## 6 Pharmacological activities of *T. cordifolia*

**Fig. 2:**
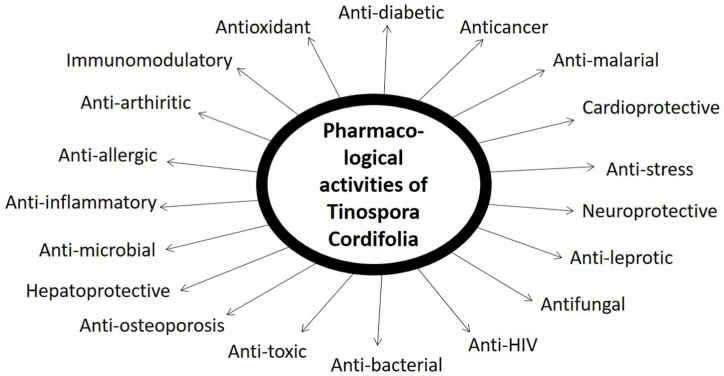
Pharmacotherapeutic potential of T. cordifolia. *T. cordifolia* has a variety of medicinal properties and therapeutic approaches in treating human health diseases and disorders such as diabetes, metabolic disorders, cardiac, hepatic, nephrotic, and neuro disorders, cancer, immune disorders, arthritis, stress, microbial, bacterial, and fungal infections, allergic reactions, and toxicity.

In the last two decades, *T. cordifolia* has been subjected to extensive scientific investigations with pharmacological importance all over the world. There are innumerable reports available for the use of *Tinospora* plant as anti-diabetic, anti-Inflammatory, antioxidant, immunomodulatory, anticancer, anti-microbial, anti-allergic, and many others (Fig. 2). Due to phytochemical substances present in *T. cordifolia* plant, such as alkaloids, phenolics, diterpenoid, glycosides, aliphatic compounds, and steroids, their pharmacological activities potentially target different diseases. Most of the pharmacological studies are based on plants’ crude extracts and biologically active compounds. In this section, we have highlighted the diverse pharmacological activities of *T. cordifolia*.

### 6.1 Anti-diabetic activity

Several pharmacological studies have clearly confirmed the antidiabetic potential of *T. cordifolia* (Table 2). Alkaloids, tannins, cardiac glycosides, flavonoids, saponins, and steroids are the major phytoconstituents reported to have an anti-diabetic role [[33], [34], [35], [36], [37], [38], [39], [40], [41], [42], [43], [44], [45], [46], [47], [48], [49], [50], [51], [52], [53], [54], [55], [56], [57], [58], [59]].

**Table 2:** Anti-diabetic potential of *T*. *cordifolia*.

Anti-diabetic studies		
Extract/isolated compounds	Animal model/Cell line/Human patient	Therapeutic outcome
Methanol	Normal and alloxan-rats	Increases in body weight and protein, hepatic enzyme hexokinase activity increased, glucose-6-phosphatase and significant decrease in fructose 1, 6-biphosphatase [33].
Isoquinoline alkaloid rich fraction	Normal and glucose-loaded Wistar rats	Insulin-mimicking and insulin-releasing effect in vitro and in vivo [34].
Hydoalcoholic (70% ethanol, 30% water)	High fat diet fed and streptozotocin-Sprague-Dawley rats	Inhibit gluconeogenesis and glycogenolysis and promote insulin secretion [35].
Aqueous, Alcoholic	Streptozotocin-albino rats	Increasing hepatic glycogen synthase and decreasing glycogen phosphorylase activity [36].
Aqueous and Alcoholic	streptozotocin diabetic albino rats	Increase in serum insulin levels or regeneration of pancreatic β cells [36].
Aqueous	Streptozotocin rats	Significant reduction in blood and urine glucose [37].
Hexane, ethyl acetate, methanol	Streptozotocin-rats	To decrease glycosylated hemoglobin level, reduce glucokinase and increased glucose-6-phosphatase activity, and to improve insulin secretagogue effect, insulin and C-peptide levels which shows β-cells regeneration capacity of extracts [38].
Aqueous	Alloxan induced diabetic rats	Significant decreases in blood glucose, glycosylated hemoglobin, urea, cholesterol, and increases in protein and glycogen, extract with nontoxic and well tolerated [39].
Aqueous	High-fructose diet induced diabetic Wistar rats	Improve glucose and lipid metabolism [40,41].
Ethyl acetate, dichloromethane, chloroform and hexane extracts	Normal and glucose-loaded Wistar rats	Reduce increased postprandial glucose level by inhibiting salivary and pancreatic amylase [42].
Aqueous extracted saponarin	Maltose-fed rats	Showed saponarin (apigenin-6-C-glucosyl-7-*O*-glucoside) with competitive inhibition on activities of alpha-glucosidase and sucrase of different origins [43].
Aqueous	Alloxan-rats	Normalized the antioxidant status of heart, brain, liver and kidney, restores the antioxidant defense [44,45].
Berberine	Clinical trial	Decrease plasma glucose and serum lipid concentrations [46].
Aqueous, Alcoholic	Streptozotocin-albino rats	Modulate renal tissue morphology and ameliorate activity of key gluconeogenic enzymes and to improve renal functions [47].
Ethanolic	Alloxan-rats	Reduce glucose level in blood [48].
Alcoholic	Alloxan-rats	Reduce glucose level in blood and urine [49].
Aqueous	Alloxan-rats	Reduce glucose level in serum [50].
Alcoholic and aqueous	Streptozotocin- mice	Amelioration of diabetic neuropathy and gastropathy [51].
Aqueous	Streptozotocin-mice	Reduce plasma glucose concentration by increasing glucose metabolism [52].
Aqueous	Alloxan-rats	Increases in body weight, total hemoglobin and hepatic hexokinase; decreases in hepatic glucose-6-phosphatase, serum acid phosphatase, alkaline phosphatase, and lactate dehydrogenase [53].
Aqueous	Alloxan-rats	Showed effect similar to 1 IU/kg of insulin [54].
Aqueous	Alloxan-rats	Reduction in serum and tissue cholesterol, phospholipids and free fatty acids [55].
Aqueous, alcoholic, chloroform	Normal and alloxan induced diabetes in rabbits	Action similar to glibenclamide and insulin [56].
Aqueous	Adrenaline induced hyperglycemia in rabbits	Significantly inhibit hyperglycemia [57].
Aqueous	Alloxan-rats and rabbits	Regulates glucose metabolism [57].
Ethanolic	Fasted albino rats	Reduce glucose level in blood [58].
Alcoholic, aqueous	Fasted albino rats	Reduce blood glucose in fasting by increasing glucose uptake and inhibition of peripheral glucose release [59].

In Asia and Africa, *T. cordifolia* has been widely used as a remedy to treat type 2 diabetes (T2D) [60]. The alkaloid rich fraction from stem; palmatine, jatrorrhizine, and magnoflorine, has been reported for insulin-mimicking and insulin-releasing effect both *in-vitro* and *in-vivo* [34]. Isolated alkaloids from the *T. cordifolia* plant showed an insulin-associated response in the induction of hypoglycemic activity [61]. A study on a diabetic model, *T. cordifolia* extract decreased high glucose, which shows anti-hyperglycemic activity [10].

Multiple sites of action of *T. cordifolia* extracts were reported, such as liver, fat, pancreatic β cells, intestinal mucosa-L cells, and muscles [62]. It also possesses multiple beneficial activities via several extra-pancreatic (primarily) and intra-pancreatic mechanisms attributed to improving the pathological status of diabetes. Its extra pancreatic activities, such as glycogenesis/inhibited glycogenolysis in the liver, improving glucose uptake and utilization, inhibiting gluconeogenesis, inhibiting intestinal glucose absorption, inhibiting α-glucoside and α-amylase, mitigating oxidative stress, antioxidant properties and protection against tissue damage, seem to contribute profoundly to diabetes [63,64]. Future research should focus on signaling pathways being affected by biologically active compound from *T. cordifolia* and consequently, effective disease targets for novel drug(s) can be identified.

### 6.2 Immunomodulatory activity

The phytoconstituents isolated from *T. cordifolia*, such as magnoflorine, tinocordioside, 11-hydroxymuskatone, cordifolioside A, N-methyl-2-pyrrolidone, and N-formylannonain, showed cytotoxic and immunomodulating activities [65]. Isolated phytoconstituents enhanced the phagocytic property of macrophages, improving nitric oxide (NO) production by stimulation of splenocytes [66] and the ability to yield reactive oxygen species (ROS) in neutrophil immune cells [67].

*T. cordifolia* treatment suppressed arthritic inflammation and bone and cartilage damage by reducing pro-inflammatory cytokines such as IL-1β, tumor necrosis factor-alpha (TNF-α), IL-6, and IL-17 [5]. Pro-inflammatory cytokine inhibition results indicated the potent activity of *T. cordifolia* against inflammatory responses. Isolated fractions (i.e., water, ethyl acetate, n-hexane, and n-butanol) and compounds (11-hydroxymustakone and N-formylannonain) exhibited splenocyte proliferation in mice [6]. The methanolic fraction of *T. cordifolia* plants caused a significant inhibition in lipo-oxygenase/cyclo-oxygenase (LOX/COX) activity and TNF-α and IL-1beta production in LPS-treated dendritic cells with moderate NO radical scavenging activity. The fraction was also found to be non-cytotoxic to monocyte cells [68].

Alcoholic and aqueous extracts of *T. cordifolia* were reported to have beneficial effects on the immune system [12]. Compound isolated from *T. cordifolia*, (1,4)-alpha-D-glucan activates the immune system through the activation of macrophages via TLR6 signaling, NF-kappaB translocation, and cytokine production in RAW cells [13]. The same isolate causes tachycardia, accompanied by hyperventilation, after intravenous administration in rats. Blood hemoglobin and hematocrit concentrations reduced significantly, but no changes were observed in respiratory variables and/or plasma inflammatory cytokine levels [69]. In an *in-vivo* study, aqueous and ethanolic extracts induced an increase in antibody production [70]. Diabetic patients with foot ulcers on *T. cordifolia* showed significantly better outcomes with improvements in wound healing [71]. Inflammatory responses are executed by a multitude of cytokines release primarily by macrophages. Phagocytosis is an essential cell defense mechanism against foreign materials, and a study on *T. cordifolia* extract showed a significant enhancement in phagocytic activity and an increase in NO and ROS [30].

Several studies have also been done that clearly show the immunomodulatory activity of *T. cordifolia* (Table 3). It is used to improve the immune system and the body's resistance against infections. The extract of *T. cordifolia* has the potential to inhibit pain and suppress inflammation due to the presence of flavonoids and alkaloids (furanolactone, tinosporin, tinosporide, jateorine, columbin, and clerodane derivatives) [80]. The methanolic extract showed good anti-inflammatory activity by inhibiting LOX enzymes and TNF-α [81]. The aqueous extract of *T. cordifolia* was found to enhance phagocytosis *in-vitro*. The chloroform extract of *T. cordifolia* inhibit the upregulation of proinflammatory biomarkers (COX-2, TNF-α, IL-6, IL-1b and iNOS) in LPS-induced RAW264.7 cells without inhibiting COX-1 [31]. *T. cordifolia* differentially regulates the elevation of cytokines, as evidenced by the increased production of anti-angiogenic agents IL-2 and tissue inhibitor of metalloprotease-1 (TIMP-1) in the B16F10-injected, extract-treated animals.

**Table 3:** Immuno-modulatory action and therapeutic outcome of *T*. *cordifolia*.

Immunomodulatory studies		
Extract/isolated compounds	Animal model/Cell line/Human patient	Therapeutic outcome
Methanolic	Dendritic cells	Significant inhibition in LOX/COX activity, TNF-α and IL-1b production in LPS-treated dendritic cells with moderate NO radical scavenging activity [68].
Methanolic	Male Lewis rats	Significant reduction of pro-inflammatory cytokines, where IL-1β, IL-6, IL-23, and TNFα and IL-17 were reduced [5].
Aqueous, n-butanol, n-hexane, Ethyl acetate. N formylannonain and 11- hydroxymustakone	Mouse splenocytes	Exhibited mouse splenocytes proliferation significantly [6].
Aqueous, n-Hexane, ethyl acetate	Polymorpho nuclear neutrophil	Increased the phagocytic activity. Ethyl acetate fraction increased the ROS and NO generation [30].
N formylann onain, N-methyl 2- pyrrolidon e, 11- hydroxymu stakone, tinocordioside, magnoflorine	Polymorpho nuclear neutrophil	Enhanced phagocytic activity of PMN and increase in nitric oxide and reactive oxygen species generation [30].
n-hexane, n-butanol, Ethyl acetate	peripheral blood mononuclear cells, PBMC	Showed good inhibitory activity in HIV-1 reverse transcriptase assay [72].
Aqueous	Peritoneal macrophages	Enhanced the nitric oxide production [66].
Aqueous	CCl4 intoxicated male albino mice	Enhanced cell adhesion and phagocytic activity. Myeloperoxidase and production also enhanced [73].
Alcoholic	Male Wister rats	Significantly increase in the WBC counts, bone marrow cells and increment in immunoglobulin [74].
(1,4)- α D Glucan	Albino Sprague Dawley Rats	Significant tachycardia without hypotension was observed. The blood hemoglobin and hematocrit concentrations also reduced significantly. No change in respiratory variables and/or plasma concentration of inflammatory cytokines was observed [69].
Polysaccharide, G1-4A	Swiss mice RAW 264.7 macrophage cell line Splenic Lymphocyte	G1-4A-induced B cell proliferation and were α degradation of IkB- also inhibited by the AntiTLR4-MD2 complex antibody. RAW 264.7 macrophages activated and enhancement in the number of CD11b + cells in the phagocytosis index in peritoneal exudate cells (PEC) [75].
Aqueous extract	Human (18–50 yrs HIV patients)	The remarkable reduction in hemoglobin percentage and eosinophil count [76].
Hydro-alcoholic	Swiss albino Strain ‘A’ Mice	Reduced apoptosis, activated macrophages and enhanced cell proliferation as well as increased level of IL-1β and GM-CSF [77].
(1,4)- α D Glucan	RAW cells	Activates the immune system through the activation of macrophages via TLR6 signaling, NF kappaB translocation and cytokine production [13].
Aqueous	Human (18–60 yrs)	Total leukocyte count increased and decreased in neutrophil and eosinophil count. Reduced all symptoms of allergic rhinitis significantly [78].
Aqueous	Wistar strain	A significant reduction of SGOT, ALP, SGPT, bilirubin in serum levels and was Increment in the functional capacities of rat peritoneal macrophages [79].

The minimum toxicity and the potential benefits of *T. cordifolia* in treating pain and inflammation suggest that it may be used in future for the treatment of these conditions. Therefore, research should be carried out on the isolation of bioactive metabolites and their proper identification to confirm their potency and efficacy in the immune system. Moreover, future investigation should be focused on phytocompounds contributing immunomodulatory and anti-inflammatory activity and correlating various signaling pathways to understand their actions at molecular and systemic levels.

### 6.3 Anti-cancer activity

The second-leading cause of death in the world is cancer. The anticancer activity of *T. cordifolia* has been reported against various tumors or cancers. Previous studies have shown that *T. cordifolia* extract arrests cells in the G0/G1 and G2/M phases by suppressing expression of the G1/S phase-specific protein cyclin D1 and the anti-apoptotic B-cell lymphoma-extra-large protein (Bcl-xL), thus supporting its anti-proliferative and apoptosis-inducing potential [28].

*T. cordifolia* extract has been shown to increase lactate hydrogenase (LDH), decrease cell viability, and increase GSH S-transferase activity *in-vitro*. Isolated polysaccharide from *T. cordifolia* has effectively reduced the metastatic potential of B16–F10 melanoma cells [82,83]. The aqueous extract of *T. cordifolia* has been shown to have potent cytotoxic activity against human colon cancer cell lines (Colo-205 and HCT-116) and lung cancer cell lines (A-549 and NCI–H322) [84]. A methanolic extract of *T. cordifolia* stem showed significant anticancer activity against MDA-MB-231 human breast cancer cell line [20].

The radioprotective role was also documented in male Swiss albino mice. *T. cordifolia* extract inhibits the harmful effects of sub-lethal gamma radiation on testes in male mice. Diterpenoid, an isolate from *T. cordifolia*, has been reported to contain chemopreventive potential in diethylnitrosamine (DEN)-induced hepatocellular carcinoma (HCC) in rats by decreasing anti-oxidant activities via sodium dismutase (SOD), catalase (CAT), and detoxification enzymes like GSH and GPx, and subsequently increasing the activities of hepatic markers such as serum glutamic oxaloacetic transaminase (SGOT), serum glutamic pyruvate transaminase (SGPT), and LDH, and decreasing serum transaminase level, thus confirming its anti-tumor effects [21,85].

*In-vivo* antiangiogenic activity of *T. cordifolia* was detected with increased levels of pro-inflammatory cytokines, including IL-1β, IL-6, TNF-α, granulocyte monocyte-colony stimulating factor (GM-CSF), and vascular endothelial cell growth factor (VEGF). Phytoconstituents 20 β-hydroxyecdysterone, Cordioside, and Columbin, isolated from *T. cordifolia*, showed significant tumor regression and survival in mice with Ehrlich ascites carcinoma [86]. Anticarcinogenic and antimutagenic activity in *T. cordifolia* extract was found in mice [87].

The toxicity of chemotherapeutic drugs sometimes creates a significant problem in the treatment of cancer using allopathy or established medicine. Various therapies using plant plant-derived products (vinblastine, vincristine, vindesine, etoposide, teniposide, paclitaxel, docetaxel, camptotecin, and irinotecan) are available [88]. The alkaloids and terpenoids (Magnoflorine, Palmatine, Tinocordiside, and Cordifolioside A) isolated from *T. cordifolia* have reported to contain anticancerous activity. Several other *in-vitro* and *in-vivo* anti-cancer studies have been done on *T. cordifolia* (Table 4). This review suggests that a detailed, focused study is needed to explore the anti-cancer potential of *T. cordifolia* and its use as a chemotherapeutic drug.

**Table 4:** Anti-cancerous activity and effective treatment response of *T*. *cordifolia*.

Anti-cancer studies		
Extract/isolated compounds	Animal model/Cell line/Human patient	Therapeutic outcome
Methanolic	MDA-MB-231	Cytotoxicity against human breast cancer cell line [20].
Palmatine	7,12-dimethylbenz(*a*)anthracene (DMBA) induced skin carcinogenes in Swiss albino mice	Significantly decrease in tumor size, number, Serum enzyme activity. Depleted levels of reduced glutathione (GSH), SOD, and catalase and increased DNA damage [21].
Aqueous	Male Swiss albino mice	Showed radioptrotective role, Amelioration of radiation-Induced Testicular Injury [65].
Dichloromethane	Ehrlich ascites carcinoma (EAC) mice	Enables tumor-free survival via depletion of GSH and glutathione-S-transferase by elevated levels of lipid peroxidation and DNA damage to tumor cells [89].
Aqueous	HeLa cells	Decreased the cell viability, increase LDH and decrease in GSH S-transferase activity [90].
Ethanolic	LNCaP cells	Stimulate the growth and proliferation of Human LNCaP cells [91].
Diterpenoid	Rats	Reported chemopreventive potential, induced hepatocellular carcinoma (HCC) by decreasing anti-oxidant activities via SOD, CAT and detoxification enzymes like GSH, GPx and subsequent increase in the activities of the hepatic markers SGOT, SGPT, LDH and decreased serum transaminase level [92].
Aqueous	Skin carcinoma mice	Increase in phase II detoxifying enzymes. Reduction of papillomas, tumor yield, tumor burden, and tumor weight [93].
Hexane	EAC mice	It blocks G1 phase of cell cycle and cause apoptosis by the formation of apoptotic bodies, nuclear condensation, and activation of caspase-3. Increased expression of pro-apoptotic gene, Bax, and decreased expression of anti-apoptotic gene, Bcl-2 [29].
Hydroalcoholic	Swiss albino mice	Showed chemopreventive role, increase in enzyme activities of cytochrome P (450) reductase, cytochrome *b*5 reductase, GST, DT-diaphorase (DTD), SOD, catalase, GPX, and GR activity in the liver [94].

### 6.4 Hepatoprotective activity

Several studies have reported in the literature that *T. cordifolia* has a protective effect against liver toxicity. Ethanolic extracts of all parts of *T. cordifolia* showed hepatoprotective activity against carbon tetrachloride-induced hepatic damage in rats [16]. A previous study on a polyherbal formulation containing *T. cordifolia* extract possessed hepatoprotective activity in CCl_4_, ethanol, and paracetamol-induced hepatotoxicity in Wister rats [95]. Another study demonstrated that aqueous extracts of *T. cordifolia* from Ayurveda Swaras and Hima significantly reduced the deleterious effect of paracetamol and exhibited significant antioxidant and hepatoprotective activities in albino mice [17,96].

*T. cordifolia* has been demonstrated to cause CCl_4_ induced liver damage and normalized liver function, as assessed and validated by biochemical liver markers (SGPT, SGOT, ALT, AST, and bilirubin) that shows its anti-hepatotoxicity property [97]. Likewise, *T. cordifolia* shows a hepatoprotective effect against alcoholism not only by lowering liver-specific enzymes and lipid levels but also by decreasing the fatty acid amides in urine [98]. Satwa prepared from three forms of *T. cordifolia* might have the potential to be used as an effective liver tonic against alcohol-induced hepatotoxicity [99]. *T. cordifolia* treatment significantly increased absorption in the intestine and reformed liver activity against alcohol-induced multivitamin deficiency [62]. In addition, *T. cordifolia* prevents anti-tubercular drugs [100] and bile salts [101] induced hepatic damage and obstructive jaundice [102]. The extract of *T. cordifolia* inactivated hepatitis B and E surface antigens as well [103].

### 6.5 Cardioprotective activity

Several animal studies on *T. cordifolia* have reported its protective role in heart disease. The root extract of *T. cordifolia* at a high dose (200 mg/kg) exerts potent cardioprotection against isoprenaline-induced cardiotoxicity in Streptozotocin (STZ) diabetic rats [104]. Dose-dependent cardioprotective activity of an alcoholic extract of *T. cordifolia* in ischemia-reperfusion-induced myocardial infarction in rats showed a reduction in infarct size and serum lipid peroxide level [105]. This cardioprotection effect may be due to its free radical scavenging activity, protecting Mg^2+^ dependent Ca^2+^-ATPase enzyme, free radical-mediated inhibition of sarcolemmal Na^+^-K^+^-ATPase activity, and Ca^2+^ channel blocking activity. Another study demonstrated the cardioprotective activity of an alcoholic extract of *T. cordifolia* in calcium chloride-induced cardiac arrhythmia in rats [106].

Stem extract of *T. cordifolia* normalizes the alteration in lipid metabolism in an STZ-induced diabetic rat model, which benefits the heart indirectly [107]. Administration of the root extract of *T. cordifolia* (2.5 and 5.0 g/kg body weight) for 6 weeks resulted in a significant reduction in serum and tissue total cholesterol, phospholipids, and free fatty acids in alloxan diabetic rats [108]. Administration of stem methanolic extract of *T. cordifolia* obviated the altered levels of enzymes (CK and LDH) and antioxidants (SOD, catalase, glutathione, and glycoproteins contents) by cadmium intoxication, suggesting *T. cordifolia* as a potent cardioprotective agent against cadmium-induced toxicity [109]. Methanolic extract of *T. cordifolia* attenuates isoprenaline-induced myocardial infarction in Wister rats, suggesting its cardioprotective activity and ability to provide strength to the membrane of the myocardium [110]. Further research should focus on the mechanistic pathways that could be affected by active compounds of *T. cordifolia* against cardiac dysfunction, hypertrophy, and heart disorders.

### 6.6 Antioxidant activity

Antioxidant plays a major role in normal physiological functions by protecting against cell damage by ROS and reducing the adverse effects of free radicals. Total flavonol and phenolic phytocompounds isolated from the formulation of the *T. cordifolia* plant showed potent antioxidant activity measured by using 1-diphenyl-2-picrylhydrazyl (DPPH) [111]. *T. cordifolia* has been reported to increase GSH levels and gamma-glutamylcysteine ligase gene expression. It also exhibited strong free radical-scavenging properties [112]. This happened because it improved the enzymatic system by controlling ROS production and normalizing the oxidative load [113].

Ethanolic extract of *T. cordifolia* showed promising antioxidant action in alloxan-induced diabetic rats that resulted in ameliorating antioxidant markers, i.e., lipid peroxidation, catalase, SOD, and GSH levels [4,114]. Few research studies found that *T. cordifolia* has been emphatic in iron-mediated lipid damage [115], enzyme induction of carcinogen and lipid peroxide inhibition in mice [94], free radical generation and lipid peroxidation during oxygen-glucose deprivation [112], and NO scavenging effects [116].

### 6.7 Anti-arthritic activity

Arthritis is characterized by chronic inflammation in the synovial membrane of affected joints that eventually leads to loss of daily function due to chronic pain and fatigue. An approximate 1% of the population suffers from rheumatic arthritis (RA), with more persistence in females than males. With the progression of the disease, patients may also have deteriorated cartilage and bone in the affected joints, which leads to permanent disability [117]. The macrophage is an important pathogenic mediator in RA, and cytokines such as TNF-α and interleukin-1 (IL-1) are the therapeutic targets. Those drugs that block TNF-α decrease joint inflammation and slow radiographic progression [118]. In addition, nonsteroidal anti-inflammatory drugs (NSAIDs) such as aspirin, disease-modifying anti-rheumatic drugs (DMARDs) like methotrexate, and immunosuppressive agents such as prednisone are widely used in the treatment of RA [119]. These drugs are quite effective, but their prolonged use may be associated with significant adverse effects such as gastrointestinal toxicity, kidney damage, or infections [120,121]. Accordingly, increasing numbers of RA patients are resorting to the use of natural herbal products [122].

An *in-vivo* study of methanol extracts of the aerial part of *T. cordifolia* on *Mycobacterium tuberculosis* in arthritic rats showed the anti-inflammatory effect of *T. cordifolia* mediated suppression of pro-inflammatory cytokines IL-1β, IL-6, IL-23, and TNF-α. However, there was not much change in the level of anti-inflammatory IL-10. Thus, *T. cordifolia* altered the balance of pro-inflammatory versus anti-inflammatory cytokines primarily by down-regulating the pro-inflammatory cytokines, particularly IL-17 and IL-1β. In addition, the ethanolic extract of *T. cordifolia* inhibited two interrelated features of arthritis: inflammation and bone damage. *T. cordifolia* ethanolic extract also induced changes in cytokines, chemokines, and mediators of bone remodeling, which play a critical role in arthritis pathogenesis [5].

A preliminary drug trial study demonstrated standardized ayurvedic polyherbal formulations to be effective and safe in controlling active RA, comparable to the treatment effect of hydroxychloroquine sulfate, a popular disease-modifying anti-rheumatic drug [123]. Another drug trial on osteoarthritis investigated the significant reduction in knee pain and improving knee function by using ayurvedic formulations [124].

### 6.8 Anti-osteoporotic activity

During aging, the loss of bone mass and strength are the most common signs and symptoms of Osteoporosis, which leads to fragility fractures. *T. cordifolia* extract in human osteoblast-like cells MG-63 and primary osteoblast cells isolated from femur of rats showed the osteoprotective effect *in-vitro* [125]. *T. cordifolia* at a dosage of 25 μg/ml stimulated the growth of osteoblasts, increased the differentiation of cells into osteoblastic lineage, and increased the mineralization of bone like matrix on both osteoblast model systems. Cell morphology studies clearly indicated the increase in cell numbers and absence of adverse change in the cell morphology after treatment with the extract.

*In-vivo* studies on rats indicated osteoprotective effect as well. Rats treated with *T. cordifolia* extract (10 mg/kg body weight) slowed down the bone loss in tibiae, as confirmed by a bone densiometric study. *T. cordifolia* extract showed estrogen-like effects in bones but not in reproductive organs like the uterus and mammary gland [126]. These findings indicate that *T. cordifolia* may be a good supplement to overcome the post-menopausal complications in women.

Abiramasundari et al. (2017) investigated the effects of an alcoholic extract of *T. cordifolia* on bone remodeling (involving osteoblastic and osteoclastic actions) *in-vitro* and protected against ovariectomy-induced bone loss *in-vivo* [127]. Elevated osteocalcin levels, increased osteogenic gene expression, and enhanced collagen deposition were all the consequences of osteoblastogenesis resulting from *T. cordifolia* extract treatment. However, its extract treatment did not have any meaningful impact on the proliferation of osteoclasts. Pretreatment with *T. cordifolia* extract at a dose of 50 mg/kg body weight/day orally for 21 days followed by treatment for 12 weeks post-ovariectomy was able to prevent ovariectomy-induced bone loss *in-vivo*. *In-vitro* studies showed that the ethanolic extract of *T. cordifolia* stimulated the proliferation of osteoblasts, but the aqueous extract of *T. cordifolia* showed no influence on cell proliferation. Study results revealed that ethanolic extract of *T. cordifolia* treatment on osteoblasts elicits pro-stimulatory effects. On the other hand, no such effect has been seen on osteoclast cells, thereby indicating that it has no effect on resorption in bone tissue [128].

### 6.9 Neurodegenerative activity

The progressive loss of structure or function of neurons and synapses leads to the death of neurons. Neurodegeneration (ND) is a composition of two words: “neuro,” referring to *nerve cells* and “degeneration,” referring to *progressive damage*. It affects millions of people worldwide. Degenerative nerve disease (DND), a group of diseases that primarily affects brain neurons, affects the body's balance, movement, talking, breathing, and heart function. Parkinson's disease, Huntington's disease, Alzheimer's disease, and spinal muscular atrophy are the common disorders of DND. These different neurodegenerative disorders lead to induced cell death as well as atypical protein assemblies [129,130].

*T. cordifolia* extract modulated the antioxidant system, such as cytosolic Cu–Zn SOD, reduced glutathione, glutathione peroxidase, and NO, and provided the neuroprotection, when the hippocampal slice was subjected to oxygen glucose deprivation [131]. Agarwal et al. (2002) studied the impact of aqueous and ethanolic extracts of *T. cordifolia* on memory enhancing property in rats [23]. Cyclosporin caused a decreased in memory, as observed by the Hebb William maze test. *T. cordifolia*, in combination with cyclosporine, successfully overcame the cyclosporine-mediated memory deficit. The histopathological examination of the hippocampus in cyclosporine-treated rats showed neurodegenerative changes, which were protected by the *T. cordifolia* plant. It also enhances cognition (learning and memory) in normal rats.

Petroleum ether extract of *T. cordifolia* showed an anti-depressant effect in rats. This effect was comparable to that of imipramine and sertraline [132]. The methanolic extract of *T. cordifolia* clearly demonstrated the actylcholinesterase inhibitory effect and improvement of cognition [22,133]. *T. cordifolia* in combination of *Phyllanthus emblica* and *Ocimum sanctum*, showed nootropic activity in normal and memory-impaired rats [134]. Aqueous extract of *T. cordifolia* was supplemented for 21 days to healthy volunteers of age 18–30 years in a double blind, randomized, and placebo-controlled study that showed a significant increase (p < 0.05) in the test scores for verbal learning and logical memory. No significant untoward effects were reported during *T. cordifolia* treatment [135].

Ethanolic extract of *T. cordifolia* enhanced the dopamine level in a 6-hydroxydopamine (6-OHDA)-induced rat model mimicking Parkinson disease. Neuroprotection was confirmed again by reduced oxidative stress and restored locomotor activity [136]. Levodopa (l-DOPA) is the most widely used drug for the treatment of Parkinson's disease. However, various studies have proved that treatment with l-DOPA leads to the death of surviving dopaminergic neurons in the central nervous system (CNS) [137]. Co-administration of l-DOPA with the crude powder of *T. cordifolia* mitigated the l-DOPA-mediated toxicity in mice [138]. As research progresses, many similarities appear that relate these diseases to one another on a sub-cellular level. Discovering these similarities offers hope for therapeutic advances that could ameliorate many diseases simultaneously.

### 6.10 Anti-stress activity

Sleep deprivation (SD) leads to a spectrum of mood disorders like anxiety, cognitive dysfunction, and motor coordination impairment. Mishra et al. (2016) studied the effect of a 50% ethanolic stem extract of *T. cordifolia* on sleep-deprived rats [139]. *T. cordifolia* extract-treated animals showed improved behavioral response in elevated plus maze (EPM) and novel object recognition (NOR) tests for anxiety and cognitive functions compared to sleep-deprived rats. *T. cordifolia* extract pretreatment modulated the stress induced-expression of plasticity markers i.e., polysialylated neuronal cell adhesion molecule (PSA-NCAM), NCAM, and growth associate protein-43 (GAP-43) along with proteins involved in the maintenance of LTP, i.e., Ca2+/calmodulin-dependent protein kinase II-α (CamKII-α), and cacineurin (CaN), in the hippocampus and PC regions of the brain [139]. *T. cordifolia* extract-treated animals showed down-regulated expression of inflammatory markers such as CD11b/c, major histocompatibility complex-1 (MHC-1), and cytokines, along with inhibition of apoptotic markers. The ethanolic extract of *T. cordifolia* showed anti-stress activity comparable to diazepalm [140]. Further studies are needed on the exact mechanism of action of the anti-anxiety agent *T. cordifolia* in enhancing memory and controlling mental stress.

### 6.11 Anti-allergic activity

*T. cordifolia* is traditionally used for the treatment of asthma, and the juice is also used for the treatment of chronic coughs [141]. Aqueous extract of *T. cordifolia* reduces mast-mediated allergic reactions in rats via anti-histaminic activities [18]. Moreover, decreased symptoms of allergic rhinitis like sneezing, nasal discharge, nasal obstruction, and nasal pruritus were also reported [78].

The aqueous extract of *T. cordifolia* stem on mast cell mediated allergic reactions *in-vivo* and *in-vitro*. *T. cordifolia* significantly inhibited the cutaneous anaphylaxis reaction activated by histamine in a rat model, and inhibition of histamine-induced contraction of the guinea-pig ileum. It significantly inhibited the secretion of TNF-α in antidinitrophenyl (DNP) IgE-stimulated rat peritoneal mast cells and also decreased intracellular calcium levels of activated mast cells. All these results showed that *T. cordifolia* may be beneficial in the treatment of acute and chronic allergic disorders [19,142]. The present review stated that *T. cordifolia* extract provides an inimitable opportunity to treat allergic disorders with little or no side effects as compared to marketed anti-allergenics.

### 6.12 Antimicrobial activity

Phytoconstituents isolated from *T. cordifolia* berberine, furanolactone, palmatine, tinosporon, jatorrhizin, and columbin-have been reported to have potential against microbial infections [143,144]. *T. cordifolia* extract has been reported to function in bacterial clearance and improve the phagocytic and intracellular bactericidal capacities of neutrophils in mice models [145]. A novel polysaccharide named G1-4A isolated from *T. cordifolia* showed inhibition against the intracellular growth of *Mycobacterium tuberculosis* through toll-like receptor 4 (TLR4)-dependent signaling [14].

The anti-bacterial activity of *T. cordifolia* extracts has been found against Bacillus *subtilis,* Escherichia *coli,* Enterobacter *aerogene,* Klebsiella *pneumoniae,* Klebsiella *pneumonia,* Micrococcus *luteus,* Proteus *vulgaris,* Pseudomonas *aeruginosa,* Pseudomonas *aeruginosa,* Salmonella *typhi,* Salmonella *paratyphi,* Salmonella *typhimurium,* Shigella *flexneri,* Serratia *marcesenses,* Staphylococcus *aureus*, and Staphylococcus *epidermidis* [14,15,146]. An aqueous extract of *T. cordifolia* showed potent antifungal activity against the fungus *Aspergillus in-vitro* [147,148]. Likewise, the ethanolic extract of *T. cordifolia* formulation used against *Streptococcus mutans* showed maximum anti-microbial activity tested *in-vitro* [149,150]. *T. cordifolia* ethanol extract also showed maximum free radical scavenging activity tested *in-vitro* [151]. Various doses of *T. cordifolia* aqueous extract administered orally to *Aspergillus* species showed a better survival rate and an effective decrease in fungal burden, *in-vivo* [148].

### 6.13 Other pharmacological activities

Other than these pharmacological activities, *T. cordifolia* studies have reported beneficial effects on disorders like leprosy, ulcers, depression, diarrhea, asthma, and infertility. The methanolic extracts from the leaves of *T. cordifolia* inhibited thrombin-induced platelet activation in rats [152].

*T. cordifolia* extract showed potency against human immunodeficiency virus (HIV) by stimulating B lymphocytes, macrophages, polymorphonuclear leucocytes, and hemoglobin percentage [9]. A moderate cytotoxic activity against peripheral blood mononuclear cells (PBMC) and good inhibitory activity against HIV-1 reverse transcriptase were recorded in n-hexane and n-butanol crude extracts [72]. Significant reductions in eosinophil count and improved hemoglobin in HIV patients were also observed [76,153].

Anti-toxic activity has been reported on the aqueous root extract of *T. cordifolia*, which showed protection against aflatoxin-induced nephrotoxicity and scavenger free radical's generation in mice's kidneys [89]. Also, it shows its ability to lower the thiobarbituric acid reactive substances (TBARS) concentration and ameliorate the antioxidant enzyme activities of SOD, GST, and GPx. Simultaneous administration of stem and root extracts of *T. cordifolia* showed protective effect against lead-induced intoxication in hematological parameters such as red blood cells and hemoglobin [154].

Due to its diversified phytocompounds and medicinal properties, *T. cordifolia* shows many biological activities. It is the most beneficial and effective plant species of *Tinospora*, which has several bioactive chemical substances present in different parts of the plant. That is the reason humans used the different parts of miraculous plant to cure various diseases and disorders since ancient times.

## 7 Coronavirus disease-19 (COVID 19) and *T. cordifolia*

In December 2019, a new type of virus emerged from Wuhan, China. The World Health Organization (WHO) named this unexplained pneumonia Coronavirus Disease-19 (COVID-19) [155]. Clinical symptoms such as dry cough, fever, sore throat, lung damage, shortness of breath, fatigue, sputum production, myalgia, and diarrhea are the symptoms present in COVID-19 patients. It is highly transmissible in humans, especially in the elderly and people with underlying diseases, due to low body immunity [156]. As of early July 2020, a total of >11.8 million cases of the Severe Acute Respiratory Syndrome Coronavirus-2 (SARS-CoV-2) infection had been reported worldwide, of which >0.74 million cases were found alone in India. Due to the rapid spread of SARS-CoV-2 through human-to-human transmission, cases are rising and then decreasing gradually. Several preclinical and clinical studies have been reported on COVID-19 since 2020 [157]. There are very few anti-corona vaccines developed and approved so far for COVID-19. Few vaccines have largely been effective against the ancestral strain of SARS-CoV-2 [158].

*T. cordifolia*, a miracle herb, in combination with other medicinal herbs, targets the target site of coronavirus in the clinical trial stage [159]. Several studies have revealed the therapeutic potential of medicinal herbs i.e., *T. cordifolia*, W. *sominfera* (Ashwagandha) and O. *sanctum* (Tulsi), and other herbs, in fighting against coronavirus disease [160]. A recent study on *T. cordifolia* and *W*. *sominifera* showed immunomodulatory potential *in-vivo* against COVID-19 [161]. Aqueous extracts of the *T. cordifolia* plant affect cytokine production and effector cells activation [162]. The person having symptoms of coronavirus consumes ‘kadha’ (decoction) of *T. cordifolia* and O. *sanctum* with *Piper nigrum* (black pepper), *Curcuma longa* (turmeric) and *Zingiber officinale* (ginger) to boost the body's immunity against the deadly disease.

In addition to herbal medicine, yoga and breathing exercises i.e., deep breathing, kapalabhati yoga, and anulom vilom pranayama, also increase body immunity and improve the respiratory system. Hence, yoga and breathing exercises work best to protect children and the elderly from the deadly coronavirus disease [163].

WHO advises people worldwide to strictly follow the government safety guidelines for COVID-19. WHO releases statements from time to time about the necessity of vaccination. Also, it is advised that people can work together for themselves and others’ safety by washing hands with soap and water and using hand sanitizer to fight against the deadly coronavirus disease [164].

## 8 Clinical relevance of *T. cordifolia*

*T. cordifolia* leaf extract was found to have a significant effect in reducing T2D [165]. Stem extract of *T. cordifolia* showed a significant decrease in fasting blood sugar, total cholesterol, triglycerides, and β-lipoproteins levels in T2D patients [166]. Likewise, the hypoglycemic potential of two ayurvedic aqueous extract formulations i.e., solidified Guduchi Ghana and sedimented starchy Guduchi Satva, has been reported in a T2D study [167]. The study result show that Guduchi Ghana is more effective than Guduchi Satva. Several other clinical studies reported on Kwatha (decoction) and Churna (fine powder) of Guduchi also support its anti-diabetic potential [97,168,169]. The herb also improved wound healing in diabetic patients with foot ulcers [71].

*T. cordifolia* showed immunosuppression in obstructive jaundice patients [102,170]. Immunomodulatory activity of herbs is reported via various mechanisms, such as increased cytokine production with macrophage activation that leads to leukocytosis and improves neutrophil function [76,171,172]. The herb also caused a significant reduction in eosinophil count and improved hemoglobin in HIV patients. In addition to that, 60% of patients showed a decrease in various symptoms associated with the disease [76,153].

A chronic alcoholism (CA) study on adult males suggested that an aqueous extract of *T. cordifolia* stem may be used either alone or in combination to reduce alcohol-induced disorders [173]. *T. cordifolia* also showed hepatoprotective and anti-stress activities as its treatment depleted the levels of SGOT and SGPT in chronic alcoholics. In addition, *T. cordifolia* shows in-vivo anti-oxidant properties because its treatment increases the levels of homocysteine and glutathione while decreasing the levels of phenyllactic acid, a non-invasive biomarker for alcoholism [173]. Increased levels of carnitine and peroxisome proliferator-activated receptors-α (PPAR-α) activation have been seen in alcoholics by the treatment of *T. cordifolia*. The same group reported that the aqueous extract of *T. cordifolia* modulates lipid metabolism by inhibiting cholesterol, triglyceride (TG), low density lipoprotein (LDL), normalized estradiol level, and significant improvement in the ratio of testosterone (T) and androstenedione (A) in plasma and urine samples of chronic alcoholics [174]. Therefore, it might also be useful in hyperlipidemic conditions. Short-term (eight-week) supplementation of a polyherbal combination (with *T. cordifolia*) drug i.e., G-400, showed a significant improvement not only for glycosylated hemoglobin but also for serum total cholesterol, HDL and LDL cholesterol, and triglycerides in T2D patients [175]. All these clinical studies have supported the safe therapeutic use of herbs as a protective agent against various diseases.

## 9 Conclusion

Despite the untreated and less effective treatment of various diseases, there has been an increase in demand for herbal medicines such as *T. cordifolia* in India, other Asian countries, and worldwide. The magical fact behind this is that herbal medicine shows effective treatment in terms of short- or long-term medication and has fewer side effects than other normal treatment therapies. Herbal medicine plays a beneficial and protective role against various diseases. This well-fitted approach to herbal medicine not only targets the ailment site but is also beneficial in boosting the immune system and improving compatibility with the human body.

The multidirectional pharmacological approaches of the *T. cordifolia* plant have been explored in the present review. Due to its therapeutic efficacy in boosting immunity, we believed that T. cordifolia could also be effective against various diseases, although researchers scientifically and doctors medically worldwide are trying to develop an effective vaccine using herbal medicine. Also, it could be one of the herbal candidates for bioprospecting and drug development for disease treatment. The presence of chemical constituents indicates that *T. cordifolia* plant could serve as a “leader in the traditional system of medicine” for the development of novel agents against diseases in the coming years. The present review provides a diversified therapeutic approach for present or future studies to carry out research on the miracle plant so that they could get medicinally important herbal drugs and use them in the treatment of various diseases. These research advances highlight the diverse pharmacological activities of *T. cordifolia*, making it a subject of continued interest in the fields of traditional medicine and modern pharmacology. Researchers are increasingly uncovering the mechanisms behind its therapeutic effects, which may lead to the development of novel treatments and therapies in the future.

## 10 Historical development and future prospects

*T. cordifolia* has a rich history of use in traditional systems of medicine, primarily Ayurveda, and has gained increasing attention in modern research. Ancient texts such as Charaka Samhita and Sushruta Samhita describe its use in treating various ailments, including fever, diabetes, skin disorders, digestive issues, and many more. It is also used in other traditional healing systems, such as Siddha and Unani medicine in India. Its medicinal properties have made it a staple in the traditional pharmacopeia of South Asian and Southeast Asian cultures. In addition to its medicinal use, *T. cordifolia* holds cultural and ritual significance in various communities. It is often associated with longevity and is considered an adaptogen or Rasayana in Ayurveda, believed to enhance vitality and longevity. In addition to that, there has been a surge in pharmacological research on *T. cordifolia*. Scientific investigations have validated many of its traditional uses and revealed its active compounds such as alkaloids, diterpenoids and polysaccharides. Herbal medicine is often considered to have minimal side effects, be less toxic, have the potential to treat several life-threatening diseases, and could provide a better alternative to allopathic medicine. The main problem with herbal medicine is that it lacks a probable mode of action. Without a mechanism of action, it would be difficult to accept the herbal formulation as a target medicine in the modern system of therapeutic application. Future works should be focused on therapeutic use of *T. cordifolia* for various diseases: i) Extensive study may explore synergistic effects when *T. cordifolia* is combined with other medicinal herbs or conventional drugs, potentially leading to more effective treatment approaches *T. cordifolia*, ii) An elaborative study is needed to understand the underlying mechanism of action to exploit the biochemical and signaling pathways of biologically active compound of *T. cordifolia* for effective disease targeting, iii) Future clinical trials should be encouraged to evaluate the therapeutic effects of isolated bioactive compounds from *T. cordifolia*, and iv) Clinical studies on *T. cordifolia* are encouraged to be performed if adequate *in-vitro*, *in-vivo* and safety data available. Further clinical trials and research studies are crucial for *T. cordifolia* to substantiate its efficacy, safety and dosage recommendations. The antioxidant and anti-aging properties of *T. cordifolia* may find application in the cosmetics and personal care Industries for skin care products. Also, become a key ingredient in nutraceuticals and dietary supplements aimed at enhancing immune function, promoting general health, and managing chronic diseases. Additionally, its adaptability and hardiness could make it valuable for agricultural and horticultural purposes to enhance crop resilience and yield. This would be necessary for broader acceptance within evidence-based medical practice. The present review suggests a scope for further research on the development of novel plant-based drugs for disease treatment, where a satisfactory cure is still not available.

## Authors’ contribution statement

AG designed the review outline. AG, PG, and GB collected the information and data from the literature, summarized the data, and drafted the manuscript. All authors have read and approved the final manuscript.

## Conflicts of interest disclosure

There is no conflict of interest to disclose.

## Ethics

None.

## Data availability statement

Data will not be required for this article.

## CRediT authorship contribution statement

**Abhishek Gupta:** Writing – review & editing, Writing – original draft, Visualization, Validation, Supervision, Software, Resources, Project administration, Investigation, Funding acquisition, Formal analysis, Data curation, Conceptualization. **Priyanka Gupta:** Writing – review & editing, Investigation, Formal analysis, Data curation, Conceptualization. **Gunjan Bajpai:** Writing – review & editing, Visualization, Validation, Resources, Investigation, Data curation, Conceptualization.

## Declaration of competing interest

The authors declare the following financial interests/personal relationships which may be considered as potential competing interests: None. If there are other authors, they declare that they have no known competing financial interests or personal relationships that could have appeared to influence the work reported in this paper.

## Figures and Tables

**figs1:**